# Therapeutic efficacy of individual head orthoses in infants with positional plagiocephaly

**DOI:** 10.1007/s00056-025-00594-x

**Published:** 2025-06-12

**Authors:** Sachin Chhatwani, Caterina Degener, Lucija Rako, Christian Kirschneck, Stephan Christian Möhlhenrich, Gholamreza Danesh, Matthias Kelker

**Affiliations:** 1 Department of Orthodontics, University of Witten/Herdecke, Alfred-Herrhausen-Str. 45, 58455 Witten, Germany; 2 Private Practice for Dentistry and Orthodontics, Heinz-Fangmann-Str. 53, 42287 Wuppertal, Germany; 3 Department of Orthodontics, University of Bonn, Welschnonnenstr. 17, 53111 Bonn, Germany; 4 Private Practice for Maxillofacial Surgery, Am Krankenhaus 4, 48231 Warendorf, Germany

**Keywords:** Cranial abnormalities, Argenta classification, Helmet therapy, Asymmetry, Active repositioning, Kraniofaziale Anomalien, Argenta-Klassifikation, Helmtherapie, Asymmetrie, Aktive Umlagerung

## Abstract

**Purpose:**

Regarding the therapy for positional plagiocephaly, a distinction is made between physiotherapeutic–osteopathic treatment and treatment using individual head orthoses.

This retrospective study aimed to compare the outcome of these treatment modalities for correcting positional plagiocephaly in infants.

**Methods:**

From an initial pool of 148 patients, two groups were matched based on age, sex, and Argenta classification. Therapy was either helmet therapy in combination with physiotherapeutic–osteopathic therapy (experimental group/95 patients) or physical therapy alone (control group/28 patients). The helmet was worn 23 h per day and adjusted if necessary. A photo-optical scan was performed pretherapeutically (T0) and posttherapeutically (T1). Besides other parameters, cephalic index (CI) and 30° diagonal difference (DD) were assessed and evaluated statistically. The mean age was 5.4 ± 1.1 months in the experimental group and 5.1 ± 1.0 months in the control group. The sex ratio in the experimental group was 61 males (64.2%) to 34 females (35.8%), and in the control group, it was 19 males (67.9%) to 9 females (32.1%).

**Results:**

After alignment of the groups, the range of correction of DD in the control group (−0.4 mm ± 2.3 mm) was lower than that in the experimental group (−4.8 mm ± 2.8 mm) which was statistically significant (*p* = 0.001). The control group presented an average CI reduction from T0 to T1 of 0.1% ± 2.1%, while the experimental group showed a significantly higher reduction of CI of 3.6% ± 3.6% (*p* < 0.001). Treatment time in the helmet therapy group averaged 2.2 ± 0.6 months, and in the control group, it averaged 1.6 ± 0.5 months (*p* < 0.001).

**Conclusions:**

In the matched groups, the reduction in CI and DD was significantly greater in the experimental group compared to the control group. Treatment with an individual head orthosis for positional plagiocephaly appears to be more effective than physical treatment alone.

## Abstract (de)

**Zielsetzung:**

Bei der Therapie der lagebedingten Plagiozephalie wird zwischen physiotherapeutisch-osteopathischer Behandlung und der Versorgung mit individuellen Kopforthesen unterschieden. Diese retrospektive Studie hatte zum Ziel, den Therapieerfolg beider Behandlungsformen im Hinblick auf die Korrektur der lagebedingten Plagiozephalie bei Säuglingen zu vergleichen.

**Methodik:**

Aus einem initialen Patientenkollektiv von 148 Kindern wurden 2 hinsichtlich Alter, Geschlecht und Argenta-Klassifikation abgeglichene Gruppen gebildet. Die Therapie bestand entweder aus einer Helmtherapie in Kombination mit physiotherapeutisch-osteopathischer Behandlung (Interventionsgruppe/95 Patienten) oder alleiniger physiotherapeutischer Behandlung (Kontrollgruppe/28 Patienten). Der Helm wurde täglich 23 h getragen und bei Bedarf angepasst. Vor Therapiebeginn (T0) sowie nach Therapieende (T1) erfolgte eine fotooptische 3D-Erfassung. Neben weiteren Parametern wurden der Cephalische Index (CI) und die diagonale Differenz bei 30° (DD) erfasst und statistisch ausgewertet. Das durchschnittliche Alter betrug in der Interventionsgruppe 5,4 ± 1,1 Monate und in der Kontrollgruppe 5,1 ± 1,0 Monate. Das Geschlechterverhältnis lag in der Interventionsgruppe bei 61 Jungen (64,2 %) zu 34 Mädchen (35,8 %) und in der Kontrollgruppe bei 19 Jungen (67,9 %) zu 9 Mädchen (32,1 %).

**Ergebnisse:**

Nach dem Abgleich der Gruppen zeigte sich eine signifikant größere Korrektur der DD in der Interventionsgruppe (−4,8 mm ± 2,8 mm) im Vergleich zur Kontrollgruppe (−0,4 mm ± 2,3 mm) (*p* = 0,001). Der CI nahm in der Kontrollgruppe von T0 bis T1 im Mittel um 0,1 % ± 2,1 % ab, während in der Interventionsgruppe eine signifikant stärkere Reduktion von 3,6 % ± 3,6 % erreicht wurde (*p* < 0,001). Die durchschnittliche Behandlungsdauer betrug in der Helmgruppe 2,2 ± 0,6 Monate und in der Kontrollgruppe 1,6 ± 0,5 Monate (*p* < 0,001).

**Schlussfolgerung:**

In den abgeglichenen Gruppen zeigten sich signifikant stärkere Verbesserungen von CI und DD in der Interventionsgruppe. Die Behandlung der lagebedingten Plagiozephalie mit einer individuellen Kopforthese erwies sich als effektiver als die alleinige physiotherapeutische Therapie.

Positional plagiocephaly is defined as an asymmetrical deformation of the occiput—which is normally shaped after birth—due to external forces [6]. Mawji et al. reported an incidence rate of 46.6% for positional plagiocephaly [18]. Possible risk factors that potentiate the development of positional plagiocephaly are neonatal intensive care, presence of torticollis [1, 4, 5], and perinatal use of suction cups or forceps [10]. Other causes include motor developmental delay [10], preferred unilateral head position [27, 38], and exclusive supine positioning of infants [1, 10, 34, 38]. To prevent plagiocephaly caused by positioning, it is necessary to recognize movement restrictions and preferred head positions at an early stage, which can be reinforced by neck muscle dysfunction. Early promotion of motor development in the sense of prone positioning (tummy time) and a reciprocal approach to the infant reduce the likelihood of developing positional plagiocephaly [1, 3, 10, 22, 29]. Possible motor developmental delays or already beginning or manifesting deformities of the infant’s skull are routinely examined after birth and at every preventive examination until the completion of the first year of life.

In the presence of positional plagiocephaly, the skull has an abnormally rounded shape and resembles more a parallelogram (Fig. 1).

**Fig. 1:**
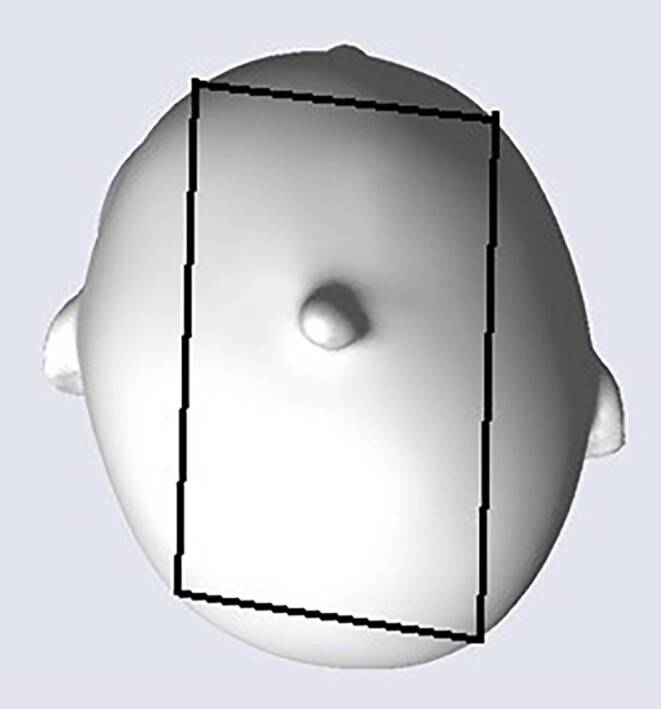
Scan of a head with positional plagiocephaly. A parallelogram-like shape is evident Scan eines Kopfes mit lagebedingter Plagiozephalie. Eine parallelogrammähnliche Form ist erkennbar

According to Argenta, the severity of positional plagiocephaly can be assessed as follows [2]:

In addition to isolated occipital flattening (type I), there may be a simultaneous ventral displacement of the skull base and one ear of the affected side (type II) and a protrusion of the forehead (type III) in the form of a parallelogram. With increasing severity, facial (type IV) and temporal or cranial (type V) deformities can be observed [2]. In addition to visual assessment, there are other metric parameters for measuring the skull, such as the cephalic index (CI) and the 30° diagonal difference (DD).

Regarding the therapy for positional plagiocephaly, a distinction is made between active repositioning techniques, physiotherapeutic–osteopathic treatment [29, 30], and treatment using individual head orthoses. Repositioning or manual therapy is initially initiated. If there is no improvement after 2 months or if the head deformity is initially moderate to severe (DD > 12 mm [23]), therapy is continued with the aid of a head orthosis. An individually manufactured helmet represents an ideal head shape with a cavity in the area of the deformity. Growth occurs through a physiological increase in the size of the neurocranium, which is promoted by growth pressure of the brain. The cavity in the helmet promotes growth in the deformities. This retrospective study aimed to assess the therapeutic efficacy of individual head orthoses combined with physiotherapeutic–osteopathic therapy (POT) in comparison to physical therapy alone for infants with positional plagiocephaly.

## Materials and methods

Data acquisition for the present retrospective study was based on the patient collective of the helmet consultation of an oral and maxillofacial surgery practice in Warendorf, Germany (2009–2015). Ethical approval (42/2013) was given by the Ethics Committee of the University of Witten/Herdecke, Germany.

Only patients with a fully completed medical history form, which included general questions, specific questions about the course of pregnancy and birth, and questions about the previous course of the disease and any previous therapy, were included. All patients underwent a comprehensive examination. This initial examination included visual examination and digital determination of the severity of the deformity using a photo-optical scan. The scan was repeated pretherapeutically (T0) and posttherapeutically (T1). Patients diagnosed with craniosynostosis were excluded from this study. As the optimal time window for severe positional plagiocephaly is in early infancy, patients over 8 months of age were also excluded from the study [35]. The patients in the control group were aged 3–7 months old at the start of therapy. A systematic alignment of the control and helmet therapy groups was performed based on age at the onset of therapy, sex, and Argenta classification. This alignment ensured comparability between the groups, allowing for a rigorous evaluation of treatment outcomes while minimizing bias related to these critical baseline characteristics.

Depending on the severity of positional plagiocephaly, therapy was either POT in combination with helmet therapy (experimental group) or POT alone (control group). The head orthosis was manufactured based on the first head scan (T0) using a thermoforming process (bagomed GmbH, Bonn, Germany), with subsequent fitting to the patient’s head and instruction of the parents regarding handling and wearing time. The helmet was worn 23 h per day after a short familiarization phase. The control intervals took place at 4‑ to 6‑week intervals with control of the helmet fit and, if necessary, an adjustment by grinding. The helmet was discontinued after the head achieved a harmonious appearance according to the subjective esthetic perception of the parents.

The duration of physical therapy in the control group averaged 4–6 weeks.

With regard to the number of patients examined, no specific sample size calculation was carried out. Instead, the present study involved a “convenient sample”, in which all available patients in a specific period were analyzed. As a consequence, the patient population in this study included 95 infants in the experimental group, of whom 61 were male and 34 female. The control group consisted of 28 infants, of whom 19 were male and 9 female.

**Fig. 2:**
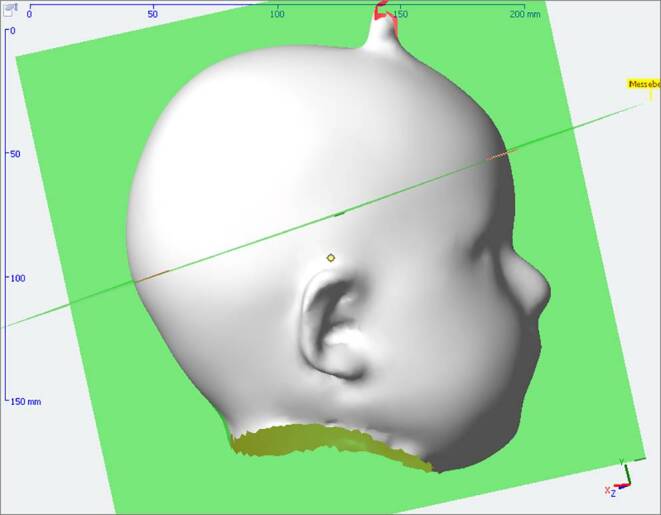
Exemplary representation of a three-dimensional head scan (taken from the software OnyxCeph, Image Instruments) showing the measurement plane Beispielhafte Darstellung eines dreidimensionalen Kopfscans (erstellt mit der Software OnyxCeph, Image Instruments) mit Anzeige der Messebene

The scan analysis was performed using an analysis tool of the OnyxCeph software (Image Instruments, Chemnitz, Germany) developed specifically for this study. The soft tissue points nasion, tragus right, and tragus left and the midpoint constructed based on the soft tissue points were used as reference points for visual representation. Based on these points, a coordinate system with an x‑axis, y‑axis, z‑axis, base plane, and measurement plane were created (Fig. 2).

**Fig. 3:**
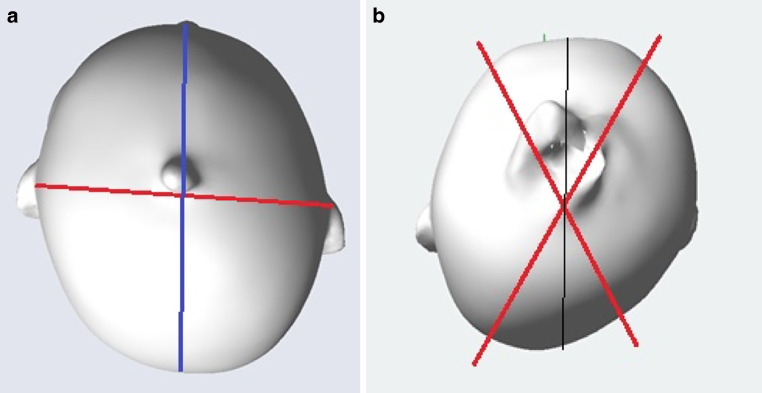
Three-dimensional scan showing maximum width (red) and maximum length (blue; **a**). Representation of the y‑axis (black) and the diagonals (red; **b**) Dreidimensionaler Scan mit Darstellung der maximalen Breite (*rot*) und maximalen Länge (*blau*; **a**). Darstellung der y‑Achse (*schwarz*) und der Diagonalen (*rot*; **b**)

Growth-related variables, such as the maximum head width and head length, were determined using the measurement plane (Fig. 3a). Using these growth-related variables, the CI (max length/max width × 100 [%]) was calculated. In the literature, different average values of the CI have been reported when measuring severity of plagiocephaly [16, 19]. The CI values in this study were based on the values used in the study by Wilbrand et al.: CI values of 81–92% for normal head shape, 92–97% for mild deformity, 97–102% for moderate deformity, and > 102% for advanced deformity [40].

The 30° DD (the difference between the longer and shorter diagonals at 30° to the y‑axis) was used as a symmetry-related variable to represent the degree of deformation (Fig. 3b). For this purpose, the shorter diagonal was subtracted from the longer diagonal, and the DD was calculated. Mild, moderate, and severe asymmetries are present for DDs of 5.1–10.0, 10.1–16.0, and > 16 mm, respectively [11].

SPSS® version 23 (IBM, Armonk, NY, USA) was used for the data analysis and interpretation.

Descriptive exploratory data analysis was performed, and the normality of the data was assessed using the Shapiro–Wilk test. Based on the normality results, nonparametric statistical methods were applied where necessary.

The differences in changes in CI and DD between the studied groups at time points T0 (pretherapeutically) and T1 (posttherapeutically) were assessed using nonparametric two-sided Mann–Whitney U test.

Regarding the parameters of sex, initial age at the start of therapy, therapy duration, and initial CI and DD values, the influence of these parameters on the success of therapy was analyzed using nonparametric methods. The Mann–Whitney U test was used to assess the influence of gender on treatment outcomes, while the Kruskal–Wallis test was used to assess the impact of initial age at the start of therapy and therapy duration on treatment outcomes.

The correlation between age at the start of therapy and therapy duration was assessed using Spearman’s rank correlation. Statistical significance was set at a *p*-value ≤ 0.05.

## Results

The descriptive statistics of the aligned groups with regard to age, sex, and Argenta classification [2] can be found in Table 1.

**Table 1:** Descriptive statistics of the matched groups with initial CI and DD values, distribution of Argenta classification and sex ratio Deskriptive Statistik der abgeglichenen Gruppen mit initialen CI- und DD-Werten, Verteilung der Argenta-Klassifikation und Geschlechterverhältnis

Parameter	Control group—mean	Control group—SD	Helmet therapy group—mean	Helmet therapy group—SD
Age in months	5.1	1.0	5.4	1.1
Initial CI in %	90.5	5.5	91.3	6.6
Initial DD in mm	13.0	3.9	14.4	4.5
Argenta distribution for matched groups				
Argenta classification	Control group (*n*)	Control group (%)	Helmet therapy group (*n*)	Helmet therapy group (%)
I	2	7.1	1	1.1
II	11	39.3	16	16.8
III	14	50.0	65	68.4
IV	1	3.6	13	13.7
V	0	0.0	0	0.0
Gender distribution				
–	Male		Female	
Control group	67.9%		32.1%	
Helmet therapy group	64.2%		35.8%	

*SD* standard deviation, *CI* cephalic index, *DD* 30° diagonal difference

The mean therapy duration in the control group was 1.6 ± 0.5 months, while in the helmet therapy group, the mean duration was 2.2 ± 0.6 months. A statistically significant difference in therapy duration was observed between the two groups (*p* < 0.001).

The mean CI value in the control group at T0 was 90.5 ± 5.5%, and at T1 it was 90.4 ± 5.7%, with a mean correction of 0.1 ± 2.1 (*p* = 0.923). The mean DD value at T0 was 13.0 ± 3.9 mm, and at T1 it was 12.6 ± 4.0 mm, with a mean correction of 0.4 ± 2.3 mm (*p* = 0.719).

In the helmet therapy group, the mean CI value at T0 was 91.3 ± 6.6%, and at T1 it was 87.8 ± 5.3%, with a mean correction of 3.6 ± 3.6% (*p* < 0.001). The mean DD value at T0 was 14.4 ± 4.5 mm, and at T1 it was 9.6 ± 3.8 mm, with a mean correction of 4.8 ± 2.8 mm (*p* < 0.001).

The intergroup comparison between the control and helmet therapy groups showed a statistically significant difference in CI correction (*p* < 0.001) and DD correction (*p* < 0.001), showing that helmet therapy resulted in greater correction for both measures as shown in Table 2.

**Table 2:** Range of correction of 30° diagonal difference (DD) and cephalic index (CI) from T0–T1 Ausmaß der Korrektur der 30°-Diagonaldifferenz (DD) und des Cephalischen Index (CI) von T0 bis T1

Group	CI	DD	Intragroup *p*-value
	Range of correction (mean ± SD)	Range of correction (mean ± SD)	
Control group	0.1 ± 2.1	0.4 ± 2.3	CI: *p* = 0.923, DD: *p* = 0.719
Helmet therapy group	3.6 ± 3.6	4.8 ± 2.8	CI: *p* < 0.001, DD: *p* < 0.001
–	Intergroup: *p* < 0.001	Intergroup: *p* < 0.001	–

*SD* standard deviation, *p p*-value

Gender had a statistically significant effect on treatment outcome in relation to CI (*p* = 0.045), with males and females showing different responses to helmet therapy. Males showed a higher reduction of CI (−4.2 ± 3.6%) than females (−2.5 ± 3.3%). However, no significant effect of gender was found on DD correction (*p* = 0.540).

The significance level for the parameter “age at therapy initiation” showed a statistically significant effect on the reduction of DD in the helmet therapy group (*p* = 0.035), indicating that earlier treatment initiation led to greater reductions in head width asymmetry. In the control group, age at therapy initiation had no significant effect on either DD or CI correction (*p* > 0.05). The greatest mean reduction in CI and DD was observed when helmet therapy was initiated before the fourth month of life (Table 3). Helmet therapy showed significantly greater corrections in both CI and DD when compared to the control group, particularly in younger patients (*p* < 0.05).

**Table 3:** Influence of age on range of correction from T0–T1 of 30° diagonal difference (DD) and cephalic index (CI) Einfluss des Alters auf das Ausmaß der Korrektur von T0 bis T1 der 30°-Diagonaldifferenz (DD) und des Cephalischen Index (CI)

Age	Control group CI	Control DD	Experimental group CI	Experimental group DD
	Range of correction (mean ± SD)	Range of correction (mean ± SD)	Range of correction (mean ± SD)	Range of correction (mean ± SD)
0–4 months	0.4 ± 2.6	1.2 ± 2.7	4.7 ± 3.9	6.1 ± 2.9
5–6 months	−0.3 ± 1.9	−0.0 ± 2.1	3.3 ± 3.6	4.6 ± 2.4
7–8 months	0.9 ± 1.8	0.1 ± 2.4	2.8 ± 2.6	3.7 ± 3.4
–	*p* = 0.319	*p* = 0.540	*p* = 0.137	*p* = 0.035

*SD* standard deviation, *p p*-value

In the control group, no significant differences were observed between the severity categories (mild, moderate, severe) for the correction of the cephalic index (CI) or the 30° diagonal difference (DD). The intragroup analysis showed a *p*-value of 0.829 for CI and 0.160 for DD, indicating that the severity of the deformity did not significantly impact the effectiveness of the physical therapy. In the helmet therapy group, no significant differences were found between severity categories for CI correction as well (*p* = 0.301). However, a highly significant difference was observed for DD correction (*p* < 0.001), with greater corrections seen in severe cases compared to mild and moderate ones. The intergroup analysis revealed significant differences between the control and helmet therapy groups for both CI and DD corrections across all severity levels. In the mild category, helmet therapy was significantly more effective than purely physical therapy for both CI (*p* = 0.028) and DD (*p* = 0.036). In moderate and severe cases, helmet therapy showed much greater improvements in CI (*p* < 0.001 and *p* = 0.015, respectively) and DD (both *p* < 0.001) corrections compared to the control group (Table 4).

**Table 4:** Influence of initial severity of plagiocephaly on range of correction from T0–T1 of 30° diagonal difference (DD) and cephalic index (CI) Einfluss des initialen Schweregrads der Plagiozephalie auf das Ausmaß der Korrektur von T0 bis T1 der 30°-Diagonaldifferenz (DD) und des Cephalischen Index (CI)

Severity	Group	CI correction (mean ± SD)	DD correction (mean ± SD)	Intragroup (CI)	Intragroup (DD)	Intergroup (CI)	Intergroup (DD)
Mild	Control	−0.1 ± 2.0	0.0 ± 1.3	*p* = 0.829	*p* = 0.160	*p* = 0.028	*p* = 0.036
	Helmet therapy	2.3 ± 2.2	2.7 ± 2.9	*p* = 0.301	*p* < 0.001		
Moderate	Control	−0.0 ± 2.3	−0.1 ± 2.6	*p* = 0.829	*p* = 0.160	*p* < 0.001	*p* < 0.001
	Helmet therapy	4.0 ± 4.2	4.0 ± 2.3	*p* = 0.301	*p* < 0.001		
Severe	Control	0.6 ± 2.2	1.7 ± 2.2	*p* = 0.829	*p* = 0.160	*p* = 0.015	*p* < 0.001
	Helmet therapy	3.3 ± 2.8	6.9 ± 2.4	*p* = 0.301	*p* < 0.001		

*SD* standard deviation, *p p*-value

## Discussion

In this study, the incidence rate of positional plagiocephaly was higher in male infants (65%), but which is slightly less than that in the study of Hutchison et al., in which 71% of the 100 patients examined with positional plagiocephaly were male [10]. A similar distribution was observed in a prospective study conducted by van Vlimmeren et al. [38]. According to them, 78% of the patients included in their study were male [38]. Sex per se is not hypothesized to play a role in the development of positional plagiocephaly, but rather in a larger head circumference and less mobility of the male infant head which might lead to higher risk of deformity during birth [17]. The effect of gender on showing a higher CI reduction in males could be explained by the larger overall skull growth in males during the investigated time period [25]. The influence of gender on DD appeared to be less pronounced, suggesting that DD and CI exhibit differential sensitivity.

Regarding the symmetry distribution of head flattening, increased flattening of the right occipital side (58%) was observed in this study. This was confirmed by the studies of Mulliken et al. (61%, [24]) and Moghaddam et al. (65%, [21]). A possible cause could be preferential unilateral head posture induced by parental carrying, feeding, or unilateral or generally decreased infant responsiveness.

Cavalier et al. reported that each immobile hour during the third and fourth months of life doubles the risk of positional plagiocephaly [3]. For this reason, according to Mortenson et al., conservative therapeutic measures before the second month of life, performed without the aid of any apparatus, serve as prevention and should be essential in the treatment of diagnosed plagiocephaly [22]. Similarly, van Wijk et al. reported that physiotherapy-assisted therapy can be useful in the treatment of positional plagiocephaly at a young age, when the deformity is less pronounced [39].

Regarding the ideal start of treatment for positional plagiocephaly, the literature often recommends starting before 12 months of age [15, 24, 29]. Kim et al. reported an ideal treatment onset at an average age of 5.6 months [12]. A similar age for treatment initiation was also found in studies by Meyer-Marcotty et al. (6.0 months) [20] and Losee et al. (7.7 months) [17]. Our findings indicate that treatment initiation should occur at an even earlier stage, as the highest range of correction was observed in the age bracket of 0–4 months. The literature often reports a total therapy duration of 4–5 months [12, 21, 32]. The duration of helmet therapy in this study was observed to be approximately 2 months shorter than previously reported in the literature, which may be attributed to the influence of early treatment initiation, wherein the reduction in CI was comparable with previous results [20]. Treatment duration was significantly shorter in the chosen control treatment group compared to the experimental treatment group (*p* < 0.001). However, the results also indicate that the control group did not perform as well as the experimental group. This finding suggests that treatment time has to be extended when applying POT alone. Treatment durations for these conservative therapies are not clearly defined, with some studies reporting a treatment period of 10 weeks and others of 5 months [8, 28].

Regarding DD and CI, Freudlsperger et al. reported that there was a positive correlation between the severity of asymmetry and the correction caused by helmet therapy [7]. This was also confirmed by the study by Kunz et al. [14]. According to them, there was a significant improvement in symmetry by helmet therapy in all age groups with moderate to severe cranial asymmetry.

A statistically significant greater reduction in DD values was observed for severe cases in the experimental groups compared to the mid and moderate cases in our study (*p* < 0.001). A possible explanation is the higher compliance and motivation of parents regarding the wearing time of the head orthosis in the presence of severe cranial asymmetry.

With respect to CI values, there was observed a trend towards more substantial reductions in the moderate and severe categories within the experimental group; however, the differences were not statistically significant (*p* > 0.05) compared to mild cases. These differing significances also indicate that the sensitivity of the DD and CI measurements differs.

Regarding the reduction of DD in the course of therapy using a head orthosis, different studies can be considered. Noto et al. investigated the effect of helmet therapy in the presence of a severe deformity by means of the parameter cranial asymmetry (CA, severe deformity CA > 12), in which the experimental group was treated by means of helmet therapy, whereas the control group received no treatment [26]. The results showed a better improvement of the plagiocephaly in the experimental group compared to that in the control group. According to them, there was no natural improvement in positional plagiocephaly in 66% of the patients in the control group (without intervention). According to Yoo et al., there is to be expected a significant improvement in the symmetry-related parameter cranial vault asymmetry (CVA) in cases with advanced initial values [41]. According to Meyer-Marcotty et al., DD was reduced by 50% (median reduction, 0.71 cm) in 20 patients with an initial value of DD > 1.2 cm (median initial value, 1.33 cm; median final value, 0.62 cm) [20]. Freudlsperger et al. reported a 43% reduction in symmetry-related parameters [7]. In our study, the experimental group exhibited a reduction of 33.3% in DD values, whereas the control group demonstrated only a 3.1% reduction. The amount of DD correction from T0 to T1 was statistically insignificant in the control group (*p* > 0.05). These findings suggest that helmet therapy was significantly more effective than POT or, alternatively, that the treatment duration may have been insufficient. It should also be taken into account that the persistence of a residual asymmetry after therapy is justified in the literature by the fact that the end of therapy was determined by the individual esthetic perception of the parents, which can be weighted as a limiting factor, and not by the presence of ideal metric parameters. Similarly, an existing asymmetry may be masked by an increase in scalp hair; thus, accurate assessment of symmetry is no longer possible. This raises the question of whether the success of therapy, and thus the duration of treatment, should be determined uniformly by metric parameters in the future or continue to be determined by the subjective feelings of the parents.

Considering CI, Teichgraeber et al. found a mean improvement in CI of 1.8% [37], whereas Meyer-Marcotty et al. observed a mean reduction of 3.7% in 20 patients [20]. In the study by Graham et al., a mean reduction in CI of 4.2% was achieved in 55 patients [9]. Thus, the mean reduction of 3.9% observed with helmet therapy in this study is consistent with the findings reported in the literature. Accordingly, helmet therapy may positively influence the CI value relative to the initial value. The moderate mean reduction in CI can be explained by the fact that helmet therapy does not reduce the size of the head but rather results in residual growth in the anterior–posterior direction to compensate for the asymmetry. Notably, the mean reduction in the control group was just 0.1% and appears negligible, which also calls into question the efficacy of this form of therapy for a short duration. A reduction of CI by pediatric manual therapy (−0.85 ± 3.63%) was also shown to be not significant when compared to a control group (−0.16 ± 2.00%) [28].

Among the studied groups, the initial DD and initial CI values were significantly reduced to a greater extent in the experimental group compared to the control group. Similar results were obtained by Kunz et al. [13] According to them, the symmetry-related parameters were significantly lower in the group with helmet therapy (*n* = 32) than in the control group (*n* = 13) [13]. The influence of a longer, purely POT approach on the reduction of symmetry-related parameters cannot be determined because of the short observation period selected for this study.

The present study has some methodological limitations. In terms of study design, this was a retrospective, nonrandomized study with consecutive patient inclusion. Data collection based on the metric parameters were retrospectively performed using three-dimensional (3D) photo-optical scans. Although 3D datasets were collected, the measurements were two-dimensional. According to Schweitzer et al., this method is the method of choice because the measurement is compliance-independent and has greater reproducibility [32]. The extent to which anthropometric measurements correlate with the assessment of the overall clinical appearance remains unclear [31]. Furthermore, the analog measurement of the metric values have been shown to deviate by 1–4 mm from the digital measurement [33], making accurate comparability of individual studies difficult. Similarly, the comparability of individual studies in this subject area is hampered by a nonstandardized methodology [36].

For these reasons, in the future, it is essential to establish a standardized methodology with regard to the examination and collection of diagnostic parameters to offer patients an objective diagnosis and an adequate and scientifically based therapy.

## Conclusion

Therapy with an individual orthosis for positional plagiocephaly in combination with physiotherapeutic–osteopathic therapy seems to be an effective measure for treatment. Reduction of the parameters examined in this study (cephalic index [CI], 30° diagonal difference [DD]) was higher compared to physiotherapeutic–osteopathic therapy alone, which does not seem to be effective when applied for a short duration. DD and CI measurements appear to exhibit varying sensitivity in assessing plagiocephaly.

## Data Availability

Data are available on request.

## Abbreviations

POT: Physiotherapeutic–osteopathic treatment
